# Morning Serum Cortisol as a Predictor for the HPA Axis Recovery in Cushing's Disease

**DOI:** 10.1155/2021/4586229

**Published:** 2021-09-03

**Authors:** Q. Cui, D. Liu, B. Xiang, Q. Sun, L. Fan, M. He, Y. Wang, X. Zhu, H. Ye

**Affiliations:** ^1^Department of Endocrinology and Metabolism, Huashan Hospital, Fudan University, Shanghai, China; ^2^Department of Neurosurgery, Huashan Hospital, Fudan University, Shanghai, China

## Abstract

**Background:**

The suppressed hypothalamic-pituitary-adrenal (HPA) axis after successful surgery for Cushing's disease (CD) will recover in almost all patients. We aimed to identify the predictive factors for HPA axis recovery in CD patients with postoperative remission. *Design and Methods*. This observational retrospective cross-sectional study enrolled 69 CD patients with postoperative remission in Huashan Hospital from 2015 to 2019. All subjects had a detailed clinical evaluation. The low-dose ACTH stimulation test (LDT) was conducted as the gold standard for assessing the HPA axis function.

**Results:**

Peak cortisol in LDT was found only to be positively correlative with morning serum cortisol (MSC) (*ρ*=0.451, *p* < 0.001). The MSC was higher (*p* < 0.001), and the median postoperative course was significantly longer (*p*=0.025) in the patients with the recovered HPA axis function compared with unrecovered patients. The AUC value of MSC for predicting the recovery of the HPA axis was 0.701, and the optimal cutoff was 6.25 *μ*g/dl (sensitivity 85.19% and specificity 47.62%). Other useful cutoff values were 10.74 *μ*g/dl (specificity 100%) and 4.18 *μ*g/dl (sensitivity 100%). Besides, combined with the postoperative course, the AUC values were higher than MSC alone (0.935 vs. 0.701, *p* < 0.001).

**Conclusions:**

MSC is a viable first-step diagnostic predictor for HPA axis recovery in CD patients with postoperative remission. For the patients with cortisol levels between 4.18 and 10.74 *μ*g/dl, a confirmatory test should be conducted. When the MSC level was 10.74 *μ*g/dl or greater, the replacement therapy could be discontinued.

## 1. Introduction

Cushing's disease (CD) is caused by pituitary adrenocorticotropic hormone- (ACTH-) secreting adenoma. It is the most common etiology of Cushing's syndrome (CS), and the optimal treatment is transsphenoidal surgery (TSS) performed by an experienced pituitary surgeon [1–3]. Typically, a transient central adrenal insufficiency (CAI) occurs after successful TSS. The patients will receive physiological hydrocortisone replacement until the suppressed hypothalamic-pituitary-adrenal (HPA) axis returns to normal function [4–6].

Besides the insulin tolerance test (ITT), which was previously considered the gold standard, the low-dose ACTH stimulation test (LDT) axis is highly correlated with the ITT. So, it could be used as a reliable test to assess the HPA axis function. However, these methods are all time and resource consuming [7–9]. The failure to detect the HPA axis recovery and an unnecessary replacement leads to complications from excess glucocorticoid administration.

In general, the tapering and discontinuation strategies for glucocorticoid replacement are controversial [3]. In the CS patient with surgical remission, clinicians were suggested to assess HPA axis recovery using the morning cortisol level every three months, followed by an ACTH stimulation test starting when the level is 7.4 *μ*g/dl (200 nmol/l) or more [3]. However, both the probability and timing of HPA axis recovery are dependent on the underlying etiology of CS [10–12].

To date, there is no specific guidance for predicting recovery of the HPA axis in the CD patients. Here, we performed this observational, retrospective, cross-sectional study to establish the predictor and the cutoff value in evaluating the recovery of HPA axis function in postoperative CD patients.

## 2. Subjects and Methods

### 2.1. Subjects

We reviewed all patients after pituitary adenoma surgery who had a LDT at the Department of Endocrinology and Metabolism of Huashan Hospital from November 2015 to November 2019. The CD patients with more than three months of laboratory-confirmed postoperative remission and a relative stable hydrocortisone replacement treatment were analyzed. Remission is defined as morning serum cortisol values below 5 *μ*g/dl (138 nmol/L) within seven days of selective tumor resection [3]. The stable replacement treatment is defined as an unchanged dosage (usually hydrocortisone 10–30 mg/day) for more than one month, without symptoms of adrenal insufficiency such as nausea, vomiting, diarrhea, and fatigue. Patients with radiation therapy or the postoperative course of fewer than three months or incomplete medical records were excluded. All subjects had a detailed clinical evaluation by the same group of endocrinology specialists. The study protocol was approved by the Research Ethics Committee of Huashan Hospital, Fudan University (No. 2017M-011).

### 2.2. ACTH Stimulation Test Protocol

A low-dose ACTH stimulation test (LDT) was conducted between 08:00 and 09:00 after an overnight fast. 0.1U (1ug) of ACTH (produced by Shanghai No. 1 Biochemical and Pharmaceutical Corp., China) was slowly injected within 2 min; blood samples for serum cortisol were collected before and 30 and 60 minutes after injection. The patients who received replacement therapy with hydrocortisone would stop the drug at least one day before the test. The normal cortisol response to ACTH stimulation was defined as the peak cortisol ≥18 *μ*g/dl (500 nmol/l) at 30 or 60 minutes. In China, ACTH is available only in vials containing 25 IU sterile lyophilized powder. A 0.1IU (1 *μ*g) dose was prepared just before administration as follows: 1 ml of sterile normal saline (NS) solution was injected into the vial, yielding a 25 IU/ml solution; then, this solution was injected into a vial containing 249 ml of NS, yielding a 0.1 IU/ml solution for administration [13].

### 2.3. Clinical and Biochemical Methods

Detailed medical history was obtained, and a complete physical examination was performed, including weight, height, and blood pressure. All tests were performed in Huashan Hospital Fudan University Laboratory. The morning serum cortisol (MSC) and ACTH were measured by electrochemiluminescence immunoassay (Modular Analytics E170-1a, Roche) following the manufacturer's instructions (A Beckman Coulter Corp.). The other blood tests including white blood cells, eosinophils, fasting blood glucose (FBG), hemoglobin A1c (HbA1c), and plasma sodium (Na^+^) were conducted by standard methods.

### 2.4. Statistical Analysis

Continuous normal data were summarized as means and standard deviations, nonnormal data are expressed as medians and interquartile ranges, and categorical variables are expressed as frequency percentage. For independent bivariate comparisons, Student's *t* and Mann–Whitney' *U* test were used according to normality. For all inferential tests, the diagnostic value of morning serum cortisol was assessed with a receiver-operated characteristic (ROC) curve and the area under the ROC curve (AUC). A value of *p* ≤ 0.05 was considered statistically significant. The optimal cutoff point of each time point was set at the closest point to the upper left corner of the ROC curve plot. The SPSS (version 19.0) was used for all of the other analyses. The ROC curve was drawn by MedCalc (version 19.1).

## 3. Results

**Figure 1:**
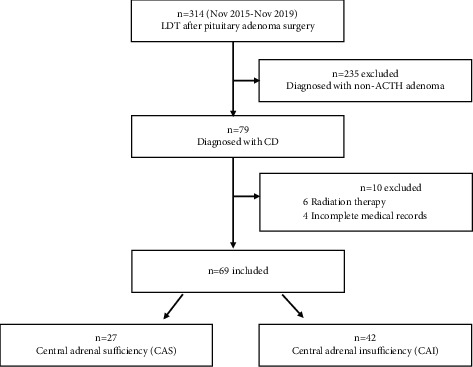
Flowchart of patient enrollment. LDT, low-dose ACTH stimulation test; ACTH, adrenocorticotropic hormone; CD, Cushing's disease.

A total of 314 patients had LDT between November 2015 and November 2019. Seventy-nine patients were pathologically diagnosed with CD. Of these, ten patients with radiation therapy or the postoperative course of fewer than three months or incomplete medical records were excluded. A total of 69 patients were included in the final analysis. By LDT, we divided the patients into the central adrenal sufficient (CAS) group (*n* = 27) and central adrenal insufficient (CAI) group (*n* = 42). A flowchart of patient recruitment is shown in Figure 1.

### 3.1. Baseline Clinical Data

Demographic and clinical characteristics are given in Table 1. The majority of the study subjects were female (90%), with a median age of 43 years old. The average values of body mass index (BMI), systolic blood pressure (SBP), diastolic blood pressure (SBP), FBG, and HbA1c in these patients are within the reference range. The median postoperative course was 11 months, and the morning serum cortisol (MSC) was 7.47 ± 2.50 *μ*g/dl, while the reference range is 6.2–19.4 *μ*g/dl in our laboratory. Of the 69 patients, 42 cases (62.9%) took replacement therapy with hydrocortisone (Table 1). More importantly, 39% of the patients had an adequate response in LDT, and of all tests, the peak cortisol level occurred at 30 minutes in 49 patients (71%) and 60 minutes in 20 patients (29%) (Table 1).

### 3.2. Comparisons between Groups with Adequate and Inadequate Response during LDT

The characteristics of the patients were compared between the CAS and CAI groups. In the CAS group, the mean concentrations of MSC were significantly higher than the CAI group (9.06 ± 2.57vs. 6.72 ± 2.17 *μ*g/dl, *p* < 0.001). In addition, the median postoperative course in the CAS and CAI groups was 16 (8, 22) months and 9 (5, 18) months, respectively, and the difference is statistically significant (*p*=0.025). No significant differences were found between the two groups in age, SBP, DBP, morning serum ACTH, serum Na^+^, FBG, HbA1c, and eosinophils ratio (EOSR) (Table 2).

Moreover, a significant positive correlation between peak cortisol in LDT and MSC was found (Spearman *ρ*=0.451, *p* < 0.001). There was no significant correlation between peak cortisol in LDT and age, SBP, DBP, morning serum ACTH, serum Na^+^, FBG, HbA1c, and EOSR.

### 3.3. The Predict Value of MSC for the Recovery of HPA Axis

**Figure 2:**
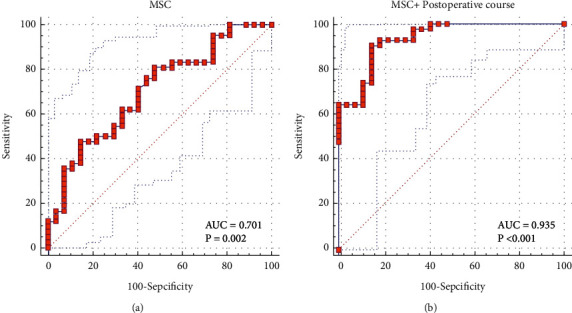
ROC curve of MSC and MSC combined with the postoperative course in the diagnosis of CAS. (a) ROC curve of MSC in the diagnosis of CAS. (b) ROC curve of MSC combined with the postoperative course in the diagnosis of CAS. ROC, receiver operating characteristics; AUC, area under the curve; CAS, central adrenal sufficiency.

The AUC value of MSC to predict CAS in LDT was 0.701 (95% CI 0.597–0.806, *p*=0.002, Figure 2(a)). Based on the Youden index, the optimal cutoff of MSC was 6.25 *μ*g/dl, which has a sensitivity of 85.19% (95% CI 66.3–95.8) and specificity of 47.62% (95% CI 32–63.3), while the positive and negative predictive values were 51.1 (95% CI 42.9–59.2) and 83.3 (95% CI 65.7–92.9%), respectively.

Besides, we found the valid cutoff values were 10.74 *μ*g/dl (specificity 100%) and 4.18 *μ*g/dl (sensitivity 100%) (Table 3), indicating that LDT should be conducted as a confirmatory test for the recovery of HPA function when the MSC level was in the range of 4.18–10.74 *μ*g/dl.

### 3.4. The Predict Value of Postoperative Course for the Recovery of HPA Axis

Correlation analysis showed the postoperative course was positively correlative with MSC (Spearman *ρ*=0.257, *p*=0.033). We found the postoperative course was another index to predict CAS in LDT, and the ROC curve analysis revealed that the AUC value was 0.661 (95% CI 0.536–0.771, *p*=0.016), and the optimal cutoff was six months.

To improve the predictive power of the MSC cutoff value in the diagnosis of CAS, we conducted the ROC curve analysis combined with MSC and the postoperative course. The results showed that the AUC value of MSC combined with the postoperative course to predict CAS in LDT was 0.935 (95% CI 0.848–0.98, *p* < 0.001, Figure 2(b)), which was significantly higher in comparison with MSC alone (*p* < 0.001).

## 4. Discussion

In this study, we confirmed the value of MSC in predicting the recovery of the HPA axis in CD patients with postoperative remission. Also, MSC combined postoperative course will significantly increase the predictive power. What is more, we found that a MSC between 4.18 *μ*g/dl and 10.74 *μ*g/dl (138–358 nmol/L) indicated that a further confirmation test, such as LDT or ITT, is needed.

In recent years, the ACTH stimulation test has been widely accepted to assess secondary adrenal insufficiency as alternatives to the ITT [3, 14–16]. In general, the low-dose ACTH stimulation test (1 *μ*g, LDT) and high-dose ACTH stimulation test (250 *μ*g, HDT) were considered to have similar diagnostic sensitivity and high specificity in CAI. It was reported that the ratio for a negative test was not suboptimal in both of the tests (LDT, 0.19; HDT, 0.39), especially in HDT, which represents a pharmacological stimulation [15]. The false-negative results may lead to premature discontinuation of cortisol replacement, which increase the risk of adrenal insufficiency in stress condition. We adopted the LDT in the study as it gives comparable and more accurate results than the HDT dose [15, 17–19]. What is more, it is reported that measuring cortisol at both 30 and 60 minutes following the ACTH stimulation test may be necessary to avoid overdiagnosing CAI [20]. Therefore, in this study, we conducted LDT in the CD patients with surgical remission. The normal cortisol response to ACTH stimulation was defined as a serum cortisol ≥18 *μ*g/dl (500 nmol/l) at 30 or 60 minutes.

The synthesis and secretion of glucocorticoids have a circadian rhythm [21, 22]. Here, as expected, MSC has a consistent correlation with the maximum cortisol response in the LDT. The AUC value of MSC for predicting the recovery of the HPA axis in LDT was 0.701, and the cutoff value that may prevent further testing was <4.18 *μ*g/dl and >10.74 *μ*g/dl. Compared with the previous studies, the predicted power was consistent [23, 24]. However, the MSC range was more valid for the need of a further confirmation test. It should be pointed out that the hypopituitarism of the subjects in the previous research literature was caused by various reasons such as pituitary tumors, intracranial lesions, surgery, and radiation therapy. In this work, all the subjects who underwent effective TSS were pathologically diagnosed with CD. The CAI was caused by the prolonged negative feedback inhibition of the normal ACTH-secreting pituitary cell due to the hypercortisolism induced by preceding pituitary adenoma [3, 25, 26]. To our knowledge, this is the first report demonstrating the predictive value of MSC in postoperative CD patients. Hurtado et al. reported that a cutoff of 10 *μ*g/dl (276 nmol/L) was routinely used in Mayo Clinic [27], which is close to the cutoff value in our study.

Moreover, we found the postoperative course was positively correlative with MSC. The postoperative course combined with MSC will significantly increase its predictive power on the HPA axis function. This result is consistent with the conclusions in the guideline for CS, that in most adults, adrenal responsiveness is restored to normal several months to a year after the operative remission. Rarely will the HPA axis fail to recover eventually [3, 11, 28].

In this study, we found the blood glucose, blood pressure, sodium, and eosinophil ratio were not significantly different between the CAI and CAS groups. This was consistent with the results of our correlation analysis, in which the peak cortisol in LDT is only related to MSC. Considering the effect of GC replacement on the electrolytes, blood glucose, and blood pressure, we analyzed the data of the 27 patients in the nonreplacement treatment group. We did not find any significant difference in FBG, HbA1C, blood pressure, blood sodium, and ESOR between the CAI and CAS patients (data not shown) either. This was supported by the result that ambulatory early morning cortisol was the only independent predictor for HPA axis recovery after prolonged glucocorticoid use [29]. On the other hand, it was reported that the patients with adrenal insufficiency, especially adrenal crisis, often have clinical features such as hypotension, hyponatremia, and hypoglycemia [8, 23, 30, 31]. As the etiology and severity of CAI are various [16, 32], we supposed that this negative result about clinical features might be because the subjects admitted to this study were postoperative CD patients who accepted routine HPA axis function evaluation. None of them had typical symptoms or complications of CAI, such as nausea, vomiting, and fatigue, infection, and fever.

There were some limitations in our study. This was a cross-sectional study, and the size of the sample is relatively small. Therefore, the conclusions should be testified by large-scaled studies, and longitudinal follow-ups were necessary in the following investigations.

## 5. Conclusions

Our results support the role of MSC as a viable first-step diagnostic test to evaluate the recovery of HPA axis function in postoperative CD patients. We suggest that for the patients with cortisol levels above 10.74 *μ*g/dl, replacement therapy could be discontinued as the HPA has recovered. When the MSC level was in the range of 4.18–10.74 *μ*g/dl, LDT should be conducted as a confirmatory test. Moreover, the postoperative course will significantly increase the diagnostic accuracy of MSC on HPA axis function. These data may help guide clinicians in the evaluation of the recovery of HPA axis function and tapering and discontinuation strategies in postoperative CD patients.

## Figures and Tables

**Table 1:** Characteristics of the patients included in the study.

Parameters	Value	Parameters	Value
No.	69	Postoperative course (m)	11 (6, 22)
Age (*y*)	43 (33, 49)	GCs relapcement	42 (61%)
Male (%)	7 (10%)	MSC (*μ*g*/*dl)	7.47 ± 2.50
Female (%)	62 (90%)	Morning ACTH (pg/ml)	23.1 (15.1, 32.5)
BMI (kg/m^2^)	22.0 (20.5, 24.8)	LDT	
SBP (mmHg)	125 ± 12	Peak in 30 min (%)	49 (71%)
DBP (mmHg)	79 ± 9	Peak in 60 min (%)	20 (29%)
FBG (mmol/L)	4.7 (4.4, 4.9)	Peak <18 (*μ*g/dl) (%)	42 (61%)
HbA1C (%)	5.4 (5.2, 6.1)	Peak >18 (*μ*g/dl) (%)	27 (39%)

Data are presented as the mean ± standard deviations, median (range), or *n* (percentage). BMI, body mass index; SBP, systolic blood pressure; DBP, diastolic blood pressure; FBG, fasting blood glucose; HbA1c, hemoglobin A1C; GCs, glucocorticoids; LDT = l.

**Table 2:** Characteristics of the patients of CAS and CAI.

Parameters	CAS (*n* = 27)	CAI (*n* = 42)	*P* value
Age (*y*)	43 (32, 54)	42 (33, 48)	0.744
BMI (kg/m^2^)	23.5 (21.0, 25.0)	21.7 (20.3, 23.0)	0.121
SBP (mmHg)	127 ± 13	123 ± 12	0.293
DBP (mmHg)	79 ± 11	80 ± 8	0.760
FBG (mmol/L)	4.8 (4.4, 4.9)	4.6 (4.3, 4.9)	0.640
HbA1C (%)	5.5 (5.2, 5.7)	5.3 (5.2, 5.7)	0.252
Na^+^ (mmol/l)	141 (139, 141)	141 (139, 142)	0.239
EOSR (%)	1.9 (1.2, 3.5)	2.7 (1.7, 3.4)	0.133
Postoperative course (m)	16 (8, 23)	9 (5, 18)	0.025^*∗*^
MSC (*μ*g*/*dl)	9.06 ± 2.57	6.72 ± 2.17	0.001^*∗*^
Morning ACTH (pg/ml)	24.2 (11.1, 33.8)	22.4 (15.2, 32.2)	0.77

^*∗*^*P* < 0.05. BMI, body mass index; SBP, systolic blood pressure; DBP, diastolic blood pressure; FBG, fasting blood glucose; HbA1c, hemoglobin A1C; EOSR, eosinophils ratio; MSC, morning serum cortisol; CAI, central adrenal insufficiency; CAS, central adrenal sufficiency.

**Table 3:** Diagnostic efficacy of MSC for the recovery of HPA axis.

Parameters	Cutoff of MSC (*μ*g/dl)		
	＞6.25	＞4.18	＞10.74
Sensitivity, % (95% CI)	85.19 (66.3–95.8)	100 (87.2–100)	18.52 (6.3–38.1)
Specificity, % (95% CI)	47.62 (32.0–63.3)	11.9 (4.0–25.6)	100 (91.6–100)
+LR (95% CI)	1.63 (1.2–2.3)	1.14 (1.0–1.3)	—
−LR (95% CI)	0.31 (0.1–0.8)	—	0.81 (0.7–1.0)
+PV (95% CI)	51.1 (42.9–59.2)	42.2 (39.5–44.9)	100
−PV (95% CI)	83.3 (65.7–92.9)	100	65.6 (61.5–69.6)

MSC, morning serum cortisol; +LR, positive likelihood ratio; −LR, negative likelihood ratio; +PV, positive predictive value; −PV, negative predictive value.

## Data Availability

The data used to support the findings of this study are included within the article. No other database was used to support this study.
